# Efficacy of a cognitive and leisure‐based training program for older adults with a memory complaint: Results of the ENGAGE trial

**DOI:** 10.1002/alz70860_104957

**Published:** 2025-12-23

**Authors:** Sylvie Belleville, Aline Moussard, Ana‐Inès Ansaldo, Patricia Belchior, Louis Bherer, Nathalie Bier, Veronique Bohbot, Lola L. Cuddy, Brigitte Gilbert, Regina Jokel, Kelly J Murphy, Natasha Rajah, Elizabeth Rochon, Jason Steffener, Angela Troyer, Nicole D. Anderson, Canadian Consortium on Neurodegeneration in Aging

**Affiliations:** ^1^ Institut Universitaire de Gériatrie de Montréal, Montréal, QC, Canada; ^2^ Department of Psychology, Université de Montréal, Montreal, QC, Canada; ^3^ IUGM research center, Montréal, QC, Canada; ^4^ University of Montreal, Montreal, QC, Canada; ^5^ McGill University, Montreal, QC, Canada; ^6^ Centre de Recherche de l'Institut Universitaire de Gériatrie de Montréal (CRIUGM), Montreal, QC, Canada; ^7^ Centre de Recherche de l'Institut Universitaire de Gériatrie de Montréal, Montréal, QC, Canada; ^8^ Montreal Heart Institute, Montréal, QC, Canada; ^9^ Research centre, Institut universitaire de gériatrie de Montréal, Montreal, QC, Canada; ^10^ Queen's University, Kingston, ON, Canada; ^11^ Institut universitaire de gériatrie de Montréal, Montreal, QC, Canada; ^12^ University of Toronto, Toronto, ON, Canada; ^13^ Baycrest Health Sciences, Toronto, ON, Canada; ^14^ Springboard Clinic, Toronto, ON, Canada; ^15^ Baycrest, Toronto, ON, Canada; ^16^ Douglas Mental Health Research Institute, Montreal, QC, Canada; ^17^ Department of Psychiatry, McGill University, Montréal, QC, Canada; ^18^ Toronto Rehab, University Health Network, Toronto, ON, Canada; ^19^ University of Ottawa, Ottawa, ON, Canada; ^20^ Baycrest Health Sciences Centre, Toronto, ON, Canada; ^21^ Rotman Research Institute, Toronto, ON, Canada

## Abstract

**Background:**

Cognitive training is recognized as an efficient approach to improve cognition in older adults at risk for dementia. Real‐world interventions that are more globally stimulating could have a greater impact on cognition than typical cognitive training programs — particularly for individuals with lower levels of education, a key risk factor for dementia. Team 10 of the Canadian Consortium on Neurodegeneration in Aging developed ENGAGE. ENGAGE is a 4‐month multifaceted program combining memory and attentional training with stimulating leisure activities. This study aimed to assess its efficacy in older adults with subjective cognitive decline or mild cognitive impairment.

**Method:**

This was a randomized controlled preference trial (trial #alz104957). One hundred and twenty‐eight participants were enrolled in two sites, CRIUGM (Montreal) and Baycrest (Toronto). There were two consecutive randomizations: a) randomization to ENGAGE‐MUSIC/SPANISH intervention vs. an active control intervention (ENGAGE‐DISCOVERY); b) randomization to SPANISH vs. MUSIC. The MUSIC and SPANISH conditions combined Music or Spanish learning with formal cognitive training. This was a preference trial, so patients could exclude Spanish or Music prior to randomization. A mixed linear model assessed PRE vs. POST changes on composite scores of memory and attention, cognition in everyday life, and use of memory strategies. Secondary analyses examined whether there were different intervention effects when comparing the MUSIC and SPANISH groups to the control intervention separately.

**Result:**

The composite score of attention and the use of memory strategies in daily life showed larger pre‐post‐training effects in the ENGAGE‐MUSIC/SPANISH intervention group than in the active control intervention, yielding a significant Intervention x Time interaction. The composite score of memory and the test of cognition in everyday life improved with time, but there was no significant interaction with intervention. Secondary analyses of the two leisure groups only identified differences in the attention composite, where SPANISH participants had a greater beneficial effect than the MUSIC participants.

**Conclusion:**

The ENGAGE program significantly improved attention and everyday memory strategy use, with differential effects of Spanish and Music. This indicates that interventions combining leisure activities with formal cognitive training have the potential for mitigating cognitive decline and reducing dementia risk.

